# Effects of Age and Gender on Hand Motion Tasks

**DOI:** 10.1155/2015/862427

**Published:** 2015-05-24

**Authors:** Wing Lok Au, Irene Soo Hoon Seah, Wei Li, Louis Chew Seng Tan

**Affiliations:** ^1^Department of Neurology, National Neuroscience Institute, Singapore 308433; ^2^Parkinson's Disease and Movement Disorders Centre, National Neuroscience Institute, NPF International Center of Excellence, Singapore; ^3^Duke-NUS Graduate Medical School, Singapore

## Abstract

*Objective*. Wearable and wireless motion sensor devices have facilitated the automated computation of speed, amplitude, and rhythm of hand motion tasks. The aim of this study is to determine if there are any biological influences on these kinematic parameters. *Methods*. 80 healthy subjects performed hand motion tasks twice for each hand, with movements measured using a wireless motion sensor device (Kinesia, Cleveland Medical Devices Inc., Cleveland, OH). Multivariate analyses were performed with age, gender, and height added into the model. *Results*. Older subjects performed poorer in finger tapping (FT) speed (*r* = 0.593, *p* < 0.001), hand-grasp (HG) speed (*r* = 0.517, *p* < 0.001), and pronation-supination (PS) speed (*r* = 0.485, *p* < 0.001). Men performed better in FT rhythm (*p* < 0.02), HG speed (*p* < 0.02), HG amplitude (*p* < 0.02), and HG rhythm (*p* < 0.05). Taller subjects performed better in the speed and amplitude components of FT (*p* < 0.02) and HG tasks (*p* < 0.02). After multivariate analyses, only age and gender emerged as significant independent factors influencing the speed but not the amplitude and rhythm components of hand motion tasks. Gender exerted an independent influence only on HG speed, with better performance in men (*p* < 0.05). *Conclusions*. Age, gender, and height are not independent factors influencing the amplitude and rhythm components of hand motion tasks. The speed component is affected by age and gender differences.

## 1. Introduction

Finger tapping and other hand motion tasks form an integral component in the motor assessment of Parkinson's disease (PD). Finger tapping (FT), hand-grasp (HG), and pronation-supination (PS) movements of the hands are used to assess bradykinesia in the upper limbs [1]. Severe PD will have slower speed, smaller amplitude, and greater variability in speed (i.e., interrupted rhythmicity) in these motor tasks. While there are specific descriptors to guide the rater in the clinical rating, for example, the Unified Parkinson's Disease Rating Scale (UPDRS) and the Movement Disorder Society-sponsored revision of the UPDRS (MDS-UPDRS) [1], the clinical rating scale is nevertheless subjective and prone to interrater and even intrarater variability. Over the years, various innovations have been developed to provide a more objective and quantitative measure for bradykinesia. Technologies such as image-based motion analysis system [2], Musical Instrument Digital Interface (MIDI) system [3], and computerised motion-sensor system have been explored [4, 5]. In recent years, wearable and wireless motion-sensor devices with automated computerized scoring system have become popular. It has been shown that these devices were more objective, reliable, and more sensitive to change than conventional clinical ratings [6].

Previous studies on hand motion tasks have shown that speed rather than amplitude responded to levodopa [7], whereas deep brain stimulation improves amplitude but not the speed of repetitive finger movements [8]. In normal subjects, the finger tapping frequency is lowered with advancing age [9, 10]. The tapping frequency is much faster in men than in women [9] and in the dominant compared to the nondominant hand [9]. However, little is known regarding the influence of age and gender on the amplitude and rhythm components of hand motion tasks.

The aim of this study is to determine if there are any biological differences with regard to the performance on speed, amplitude, and rhythm for each of the hand motion tasks (FT, HG, and PS) in a normal population.

## 2. Methods

We recruited 80 healthy subjects for the study. All were right-handed, aged 21-years and above (mean age 48.8 ± 17.9 years, range 21.0–83.6 years), without disorders of the central or peripheral nervous system, and without significant joint or bone problems that may interfere with their limb mobility. Information such as age, gender, ethnicity, and height was obtained. Handedness was established through self-report by the subjects of their preferential hand for writing and performance of daily activities. Subjects with left-handedness and mixed-handedness and those with switch of handedness were excluded from the study. Subjects performed the hand motion tasks (FT, HG, and PS) as described in the UPDRS. The movements were quantified using Kinesia (Cleveland Medical Devices Inc., Cleveland, OH), a commercial assessment device with gyroscope and accelerometer technology. For each of the speed, amplitude, and rhythm components of hand motion tasks (FT, HG, and PS), Kinesia converts the gyroscope and accelerometer data into a 0 to 4 scale of increasing severity, with 0.1 resolutions. The zero score is based on a predetermined cut-off value for the accelerometer and gyroscope data. The Kinesia scores correlated with the Modified Bradykinesia Rating Scale (MBRS) for PD and had greater test-retest reliability and sensitivity to change than conventional clinical rating scales such as the UPDRS and MBRS [6]. Subjects performed the hand motion tasks twice for each hand. The intraclass correlation coefficient (ICC) was calculated to determine the test-retest reliability for each kinematic parameter. The Kinesia scores across both hands were then averaged to obtain the mean value for each kinematic parameter. Multivariate analyses (with age, gender, and height added into the model) were performed to look for possible independent factors influencing these parameters. All subjects gave written informed consent to the study. The study was approved by the Institution Ethics and Review Board.

## 3. Results

80 subjects were recruited (39 men, 41 women). The majority were ethnic Chinese (95% ethnic Chinese, 0% ethnic Malays, 2.5% ethnic Indians, and 2.5% others). There was no significant difference in the mean age between men (50.1 ± 17.3 years) and women (48.8 ± 17.1 years). Men were taller than women (men: 170.9 ± 8.3 cm, women: 157.4 ± 4.8 cm, *p* < 0.001). There was a significant age-height interaction—decreasing height with advancing age (*r* = −0.25, *p* < 0.03). The mean UPDRS motor score for the 80 healthy subjects was 0.5 ± 1.1. For the dominant hand, the subscores for FT, HG, and PS were as follows: 0.03 ± 0.16, 0, 0.01 ± 0.11. For the nondominant hand, the corresponding subscores were 0.08 ± 0.31, 0.03 ± 0.16, and 0.038 ± 0.25.

Table 1 shows the mean Kinesia scores for the various kinematic parameters and their respective ICC. The mean scores were less than 1.0 for most tasks, except for FT_Amplitude and HG_Amplitude where the scores were approximately 1.5. The ICC was good (≥0.8) for all parameters except the rhythm component of the FT, HG, and PS tasks (≤0.6). There was a trend towards better performance in the dominant hand for the speed and rhythm component of hand motion tasks, and a poorer performance in the amplitude. The* t*-tests reached statistical significance in the following parameters: FT_Speed (*p* < 0.05), PS_Speed (*p* < 0.05), HG_Amplitude (*p* < 0.0005), and PS_Amplitude (*p* < 0.0001).

Table 2 shows the Kinesia scores stratified by gender and hand dominance. The differences in Kinesia scores between the dominant and the nondominant hand were observed within each gender group, reaching statistical significance mainly in the HG and PS tasks. Comparing gender groups, the scores were higher in women than in men for the FT and HG tasks, but lower in women than in men for the PS tasks. The* t*-tests reached statistical significance in the following parameters: FT_Rhythm in the nondominant hand (*p* < 0.05), HG_Speed in the dominant hand (*p* < 0.01), HG_Amplitude in the dominant hand (*p* < 0.02), HG_Amplitude in the nondominant hand (*p* < 0.05), HG_Rhythm in the dominant hand (*p* < 0.05), and PS_Amplitude in the nondominant hand (*p* < 0.05). Taking the mean score across both hands, men had lower scores than women in FT_Rhythm (men: 0.74 ± 0.13, women: 0.83 ± 0.17, *p* < 0.02), HG_Speed (men: 0.81 ± 0.35, women: 1.00 ± 0.30, *p* < 0.02), HG_Amplitude (men: 1.35 ± 0.43, women: 1.60 ± 0.40, *p* < 0.02), and HG_Rhythm (men: 0.65 ± 0.14, women: 0.72 ± 0.15, *p* < 0.05). The remaining kinematic parameters were not significantly different between men and women.

Table 3 shows the correlation coefficients between age and the speed component of FT, HG, and PS tasks. Overall, there were significant correlations in the positive direction in both gender groups. The mean Kinesia scores across both hands increased with age in the speed component of FT (*r* = 0.593, *p* < 0.001), HG (*r* = 0.517, *p* < 0.001), and PS tasks (*r* = 0.485, *p* < 0.001). The amplitude and the rhythm component of FT, HG, and PS tasks showed no significant correlations with age, with the exception of PS_Amplitude where the correlation was statistically significant but weak (*r* = 0.271, *p* < 0.01). Subgroup analysis showed the correlation was significant only in the dominant hand amongst women (*r* = 0.357, *p* < 0.05).

The correlations between Kinesia scores and height were statistically significant but weak. The scores decreased with increasing height in FT_Speed (*r* = −0.298, *p* < 0.01), FT_Amplitude (*r* = −0.240, *p* < 0.02), HG_Speed (*r* = −0.322, *p* < 0.01), and HG_Amplitude (*r* = −0.232, *p* < 0.02).

Multivariate analysis with age, gender, and height added into the model showed an independent effect of age on the speed component of all hand motion tasks (*p* < 0.001). There was an independent effect of gender on the speed component of HG tasks only, with slower speed in women than in men (*p* < 0.05). Height was no longer an independent factor influencing the speed, amplitude, and rhythm components of hand motion tasks. The effects of age and gender on the hand motion tasks may be expressed by the following equations:

(1) FT_Speed=0.013∗Age  years+0.306,HG_Speed=0.01∗Age  years+0.2∗Gender  men=1,women=2+0.088,PS_Speed=0.008∗Age  years−0.168.

**Figure 1:**
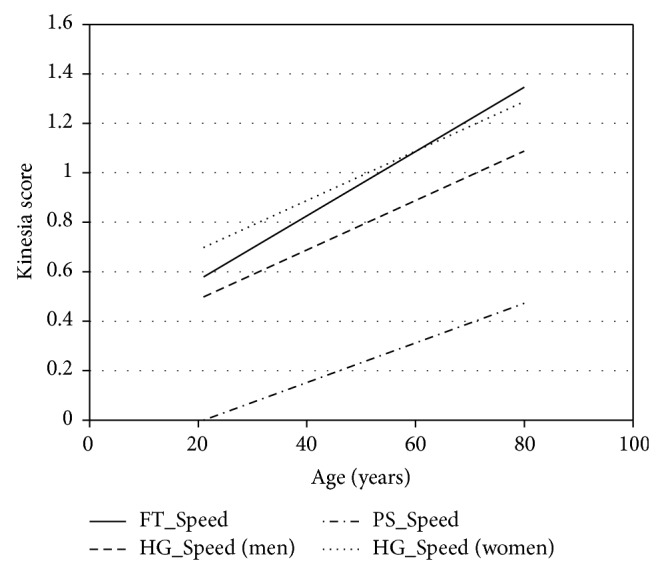
Regression plot of Kinesia scores on age for the speed component of FT, HG, and PS tasks. The multivariate equations were as represented in (1).

Figure 1 shows the regression plots of Kinesia scores on age.

## 4. Discussion

Our study showed a discrepancy between the Kinesia scores and the clinical rating scores for the various hand motion tasks in a normal population. While the clinical rating scores were closer to a score of 0, the Kinesia scores were closer to a score of 1.0 in most tasks and to 1.5 in the amplitude components of FT and HG tasks. We believe this discrepancy was due to the sensitivity of Kinesia in detecting changes up to 0.1 resolutions [6], as opposed to the subjective clinical rating scale which is a 5-point ordinal scale ranging from 0 to 4. Hence performance of hand motion tasks with a Kinesia score of 1.0 to 1.5 may be within normal limits. Amongst the different hand motion tasks, PS provides Kinesia scores closer to a score of 0 than FT and HG tasks.

The test-retest reliability was very good for the speed and amplitude components of hand motion tasks, but not for the rhythm component. This finding suggests highly variable rhythmicity of movements even amongst healthy individuals, possibly due to fatigue with fast tapping [11]. In monitoring disease progression and treatment response, it may be advisable to monitor the speed and amplitude components rather than the rhythm component of hand motion tasks. Nevertheless, one should be aware of the frequency-amplitude tradeoff [11]. When comparing the dominant versus the nondominant hand, we noticed a better performance in the dominant hand for the speed and rhythm components of hand motion tasks, at the expense of smaller amplitudes. With fast tapping, there is a tendency towards short-amplitude movements [11].

Previous studies have shown a slower finger tapping rate with advancing age [9, 10] and with men tapping faster than women [9]. In our study, after multivariate analyses, only age and gender emerged as significant independent factors influencing the kinematic parameters. In particular, only the speed component was affected, but not the amplitude and rhythm components. Gender exerted an independent influence only on the speed component of HG tasks.

There are limitations to our study. We recruited mainly right-handed ethnic Chinese which may not be generalized to other populations. Left-handedness may be under reported amongst ethnic Chinese due to cultural and practical considerations [12]. As such, we have excluded individuals with left-handedness and mixed-handedness and those with a switch of handedness and included only individuals who consistently use their right hand for writing and when performing other activities of daily living such as brushing of teeth, feeding, and use of common household tools. We did not have information on the occupations, sports, and leisure activities in our subjects. These activities may have an association with handedness and may affect performance of motor tasks [13–15]. Subjects were asked to tap as fast and as wide as possible, which may not be valid and reliable in detecting alterations in rhythm formation [11]. Nonetheless, the use of a wearable and wireless motion sensor device had facilitated a more objective measurement of the speed, amplitude, and rhythm components of hand motion tasks.

## 5. Conclusions

In an Asian population comprising mainly right-handed ethnic Chinese, age, gender, and height are not independent factors influencing the amplitude and rhythm components of hand motion tasks. The speed component is affected by age and gender differences. Further studies are needed to evaluate the performance of hand motion tasks amongst left-handed individuals and those with mixed- or switch-handedness.

## Figures and Tables

**Table 1:** Kinesia scores and ICC for the speed, amplitude, and rhythm components of hand motion tasks (FT, HG, and PS).

TASKS	DH		NDH		BH
	Mean score (mean ± SD)	ICC, 95% CI, *p* value	Mean score (mean ± SD)	ICC, 95% CI, *p* value	Mean score (mean ± SD)
FT_Speed	0.90 ± 0.40	0.867, 0.792–0.914, *p* < 0.001	0.97 ± 0.42	0.915, 0.867–0.945, *p* < 0.001	0.93 ± 0.39

FT_Amplitude	1.58 ± 0.50	0.845, 0.759–0.901, *p* < 0.001	1.50 ± 0.51	0.831, 0.736–0.891, *p* < 0.001	1.54 ± 0.47

FT_Rhythm	0.76 ± 0.20	0.240, −0.185–0.513, *p* = NS	0.79 ± 0.22	0.441, 0.128–0.642, *p* < 0.01	0.77 ± 0.18

HG_Speed	0.87 ± 0.38	0.952, 0.925–0.969, *p* < 0.001	0.91 ± 0.37	0.896, 0.838–0.933, *p* < 0.001	0.89 ± 0.35

HG_Amplitude	1.53 ± 0.48	0.906, 0.853–0.940, *p* < 0.001	1.39 ± 0.50	0.882, 0.816–0.924, *p* < 0.001	1.46 ± 0.46

HG_Rhythm	0.67 ± 0.17	0.582, 0.348–0.732, *p* < 0.001	0.69 ± 0.22	0.591, 0.362–0.738, *p* < 0.001	0.68 ± 0.17

PS_Speed	0.17 ± 0.28	0.865, 0.789–0.913, *p* < 0.001	0.23 ± 0.31	0.813, 0.709–0.880, *p* < 0.001	0.20 ± 0.27

PS_Amplitude	0.98 ± 0.38	0.887, 0.824–0.928, *p* < 0.001	0.81 ± 0.40	0.774, 0.648–0.855, *p* < 0.001	0.90 ± 0.35

PS_Rhythm	0.45 ± 0.25	0.623, 0.413–0.758, *p* < 0.001	0.49 ± 0.28	0.536, 0.276–0.702, *p* < 0.001	0.47 ± 0.21

DH = dominant hand.

NDH = nondominant hand.

BH = average of both hands.

NS = not statistically significant.

FT = finger tapping.

HG = hand-grasp.

PS = pronation-supination.

**Table 2:** Kinesia scores stratified by gender and hand dominance.

Tasks	Men (*n* = 39)			Women (*n* = 41)		
	DH (mean score ± SD)	NDH (mean score ± SD)	*p* value	DH (mean score ± SD)	NDH (mean score ± SD)	*p* value
FT_Speed	0.84 ± 0.37	0.91 ± 0.42	NS	0.99 ± 0.39	1.04 ± 0.40	NS
FT_Amplitude	1.50 ± 0.48	1.43 ± 0.52	NS	1.69 ± 0.44	1.60 ± 0.44	NS
FT_Rhythm	0.74 ± 0.16	0.75 ± 0.19	NS	0.81 ± 0.20	0.84 ± 0.21	NS

HG_Speed	0.77 ± 0.39	0.85 ± 0.35	<0.05	1.00 ± 0.31	0.99 ± 0.34	NS
HG_Amplitude	1.43 ± 0.45	1.28 ± 0.48	<0.01	1.67 ± 0.42	1.52 ± 0.45	<0.01
HG_Rhythm	0.64 ± 0.13	0.67 ± 0.21	NS	0.71 ± 0.17	0.73 ± 0.20	NS

PS_Speed	0.21 ± 0.30	0.25 ± 0.32	NS	0.14 ± 0.27	0.22 ± 0.30	0.05
PS_Amplitude	1.09 ± 0.33	0.88 ± 0.40	<0.0001	0.91 ± 0.38	0.77 ± 0.38	<0.05
PS_Rhythm	0.45 ± 0.29	0.51 ± 0.29	NS	0.46 ± 0.20	0.48 ± 0.27	NS

DH = dominant hand.

NDH = nondominant hand.

NS = not statistically significant.

**Table 3:** Correlation coefficients between age and the speed component of FT, HG, and PS tasks.

	FT_Speed	HG_Speed	PS_Speed
Men			
DH	*r* = 0.472, *p* < 0.01	*r* = 0.567, *p* < 0.001	*r* = 0.520, *p* < 0.001
NDH	*r* = 0.543, *p* < 0.0005	*r* = 0.573, *p* < 0.0002	*r* = 0.370, *p* < 0.05
Women			
DH	*r* = 0.594, *p* < 0.0001	*r* = 0.367, *p* < 0.05	*r* = 0.515, *p* < 0.001
NDH	*r* = 0.663, *p* < 0.0001	*r* = 0.535, *p* < 0.0005	*r* = 0.357, *p* < 0.05

DH = dominant hand.

NDH = nondominant hand.
